# Can an Incremental Step Test Be Used for Maximal Lactate Steady State Determination in Swimming? Clues for Practice

**DOI:** 10.3390/ijerph18020477

**Published:** 2021-01-08

**Authors:** Mário C. Espada, Francisco B. Alves, Dália Curto, Cátia C. Ferreira, Fernando J. Santos, Dalton M. Pessôa-Filho, Joana F. Reis

**Affiliations:** 1 Polytechnic Institute of Setúbal, Department of Science and Technology, 2914-514 Setubal, Portugal; mario.espada@ese.ips.pt (M.C.E.); catia.ferreira@ese.ips.pt (C.C.F.); fernando.santos@ese.ips.pt (F.J.S.); 2 Quality of Life Research Centre, 2040-413 Rio Maior, Portugal; 3 Faculdade de Motricidade Humana, Universidade de Lisboa, 1499-002 Cruz Quebrada-Dafundo, Portugal; falves@fmh.ulisboa.pt (F.B.A.); daliacurto@fmh.ulisboa.pt (D.C.); 4 Interdisciplinary Center for the Study of Human Performance (CIPER), Faculdade de Motricidade Humana, Universidade de Lisboa, 1499-002 Cruz Quebrada-Dafundo, Portugal; 5 Training Optimization and Sports Performance Research Group (GOERD), Faculty of Sport Science, University of Extremadura, 10003 Cáceres, Spain; 6 Department of Physical Education, São Paulo State University (UNESP), Bauru 17033-360, Brazil; dalton.pessoa-filho@unesp.br; 7 Institute of Bioscience, Graduate Program in Human Development and Technology, São Paulo State University (UNESP), Rio Claro 13506-900, Brazil

**Keywords:** well-trained swimmers, maximal lactate steady state, lactate threshold, continuous test, incremental test, performance markers

## Abstract

We aimed to compare the velocity, physiological responses, and stroke mechanics between the lactate parameters determined in an incremental step test (IST) and maximal lactate steady state (MLSS). Fourteen well-trained male swimmers (16.8 ± 2.8 years) were timed for 400 m and 200 m (T_200_). Afterwards, a 7 × 200-m front-crawl IST was performed. Swimming velocity, heart rate (HR), blood lactate concentration (BLC), stroke mechanics, and rate of perceived exertion (RPE) were measured throughout the IST and in the 30-min continuous test (CT) bouts for MLSS determination. Swimming velocities at lactate threshold determined with log-log methodology (1.34 ± 0.06 m∙s^−1^) and Dmax methodology (1.40 ± 0.06 m∙s^−1^); and also, the velocity at BLC of 4 mmol∙L^−1^ (1.36 ± 0.07) were not significantly different from MLSSv, however, Bland–Altman analysis showed wide limits of agreement and the concordance correlation coefficient showed poor strength of agreement between the aforementioned parameters which precludes their interchangeable use. Stroke mechanics, HR, RPE, and BLC in MLSSv were not significantly different from the fourth repetition of IST (85% of T_200_), which by itself can provide useful support to daily practice of well-trained swimmers. Nevertheless, the determination of MLSS_v_, based on a CT, remains more accurate for exercise evaluation and prescription.

## 1. Introduction

Performance enhancement in sport is closely and decisively related to accuracy in identifying exercise intensities domains toward the optimization of daily training. Specifically, the determination of the boundaries (thresholds) that separate the individual training zones can induce optimal adaptations in athletes and should be periodically assessed to evaluate training effects [1]. Over the years, this has been challenging for athletes, coaches, and researchers largely because the time spent in testing procedures may interfere with the training routines, and fundamentally, the high costs with equipment prevents its widespread use. Nevertheless, the combination of high accuracy with less time demanding or invasive procedures sustains the drive for continuing research on the applied physiology of sports training [2].

Lactate threshold (LT) was originally defined as the onset of blood lactate above resting values from a blood lactate velocity curve obtained in an incremental step test (IST) [3]. It represents the first increase of a metabolic acidosis and oxidative capacity of athletes and is a strong determinant of performance within populations of similar maximum oxygen uptake [4]. It is also an important reference point when setting training intensities for endurance athletes, having previously been considered by some researchers as a good surrogate of maximal lactate steady state (MLSS) [5], which by itself represents not only a boundary intensity between the heavy and severe intensity domains but also a biomechanical boundary beyond which stoke length (SL) becomes compromised over time [6]. However, multiple definitions and methods to determine LT have arisen in the literature. It has been considered as the initial rise in BLC above rest, the onset of a fixed blood lactate accumulation ranging from 2.0 to 4.0 mmol L^−1^, or with curve fitting procedures [7]. These methods seem to be correlated, but their relationship with the boundaries that separate the intensity domains are affected by the methodology chosen, without a clear physiological support for the preference of one of them [8].

The swimming velocity at LT (vLT) is one of the most frequently used indices to assess the swimming endurance capacity [9,10] and several methods are utilized for its calculation [11]. More specifically, the log-log methodology (vLT_log-log_), the velocity at which lactate increased exponentially when the log blood lactate concentration (BLC) is plotted against the log swimming velocity [12], Dmax methodology (vLT_Dmax_) as the maximal perpendicular distance of the lactate curve from the line connecting the start with the endpoint of the lactate curve [13]. On the other hand, V_4_ is the the swimming velocity eliciting a lactate concentration of 4 mmol L^−1^ through linear interpolation and has been associated with the onset of blood lactate accumulation (OBLA) [14]. More specifically, a 7 × 200-m IST is used to identify aerobic training intensity domains and subsequent changes during a year-round training plan [15].

Heck et al. [5] were pioneers with respect to the MLSS concept, first considered to occur at a fixed BLC of 2.2 mmol L^−1^ [16], but more often to 4 mmol L^−1^, or defined as OBLA [5]. Later, it was observed that the absolute BLC at MLSS velocity (MLSSv) varied considerably between individuals and between exercise modalities [17]. However, it is considered to be the best predictor of aerobic endurance performance [18], defined as the highest constant exercise intensity that can be sustained while maintaining equilibrium between the processes of blood lactate accumulation and elimination [1]. It is noteworthy that very recently Jones et al. [19] outlined concerns with the arbitrariness of the definition of, and the procedures for evaluating MLSS, indicating that progress in these fields has been slowed by the disagreement over definitions and procedures, and by a fixation with the behavior of a single biomarker, BLC.

The methodology associated with MLSS determination separates it from most other lactate parameters. The major methodological difference is that it requires several exercise bouts of longer duration (30-min) to be performed in different days at a constant exercise intensity, a constant intensity test (CT), a method that is very time-consuming and demanding. MLSS is attained when, in a CT lasting at least 30-min, the BLC does not increase more than 1.0 mmol L^−1^ after the 10th testing minute [20] and Billat et al. [18] stated that to achieve a true MLSS it is necessary to have four or five prolonged exercise sessions of up to 30-min duration.

Over the last 10 years, studies related to MLSS in swimming are scarce, namely those using unimpeded swimming (without oxygen uptake breath by breath apparatus), and the large majority present MLSSv below 1.30 m∙s^−1^, which can preclude the application of those results to well-trained or high-level swimmers. Some examples are studies with twelve adult middle-distance and long-distance male swimmers which found a MLSSv of 1.22 ± 0.05 m∙s^−1^ [6], with ten male swimmers and a MLSSv of 1.17 ± 0.11 m∙s^−1^ [21] and with seventeen long-distance swimmers with a MLSSv of 1.09 ± 0.14 m∙s^−1^ [9]. More recently, evaluating twenty well-trained competitive swimmers MLSSv was determined at 1.29 ± 0.05 m∙s^−1^ [22] and Nikitakis et al. [23] observed that MLSSv was higher in adolescents compared to children (1.297 ± 0.056 m∙s^−1^ vs. 1.083 ± 0.065 m∙s^−1^).

To overtake the time-consuming limitations of the CT, researchers developed an attempt to determine MLSS from a BLC curve obtained during a swimming IST [11,24] fact that is somewhat controversial in the scientific community, namely the interchangeably use of ISTs and CTs to provide useful indexes of aerobic potential [25]. Despite LT and MLSS being regularly considered fundamental physiological concepts in sport [17] most of the studies were conducted in the laboratory using ergometers in the context of running and cycling [26]. Therefore, research in swimming is scarce compared to other sports because of the swimming pool constrains. In an individual sport where results and medals are regularly decided by hundredths of a second, the accuracy of training prescription becomes extremely relevant. Additionally, from a practical point of view, swim coaches and sport scientists require accurate methods that allow them to evaluate the progress of their swimmers and to fine point the training prescription with minimal training time interference. Thus, it is necessary to have an in-depth understanding of assessment protocols and associated swimmer responses, namely from a physiological and stroke mechanics perspective in order to select the most suitable evaluation protocols and interpret their results.

Therefore, the purpose of this study is to determine if the velocities associated with the lactate parameters determined from a single IST is equivalent to MLSSv determined from several CT. Additionally, we intent to ascertain if the stroke and physiological parameters, representative of MLSS, are similar to those obtained during the IST.

## 2. Materials and Methods

### 2.1. Study Design

**Figure 1:**
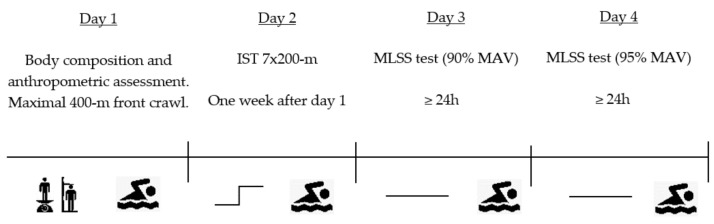
Schematic representation of the experimental protocol. IST, incremental step test; MLSS, maximal lactate steady state test (continuous test); MAV, maximal aerobic velocity.

Athletes performed a total of four visits to the water training facility within a 10-day period. On the first visit, all athletes provided written consent to participate in this study, as well as performed an anthropometric and body composition evaluation. Afterwards, the athletes performed a maximal 400-m front crawl (T_400_) in order to use the average velocity between 50 and the 350 m as an estimate of the maximal aerobic velocity (MAV) [27]. After one week, swimmers performed the IST. In days 3 and 4, the continuous swimming velocity MLSS tests (CT) were completed, first at 90% of MAV and in day 4 at 95% of MAV. Figure 1 illustrates the experimental protocol.

### 2.2. Participants

Fourteen male competitive swimmers volunteered for this study (mean ± SD; 16.8 ± 2.8 years, 1.78 ± 0.05 m, 66.5 ± 7.2 kg and 10.2 ± 2.6% of body fat). The inclusion criteria were: (1) regularly competing at national level for at least three years and (2) time in 400-m front crawl below 4:35-s. The exclusion criteria were: (1) swimmers with <14 years of age; and (2) swimmers injured three months before experimental protocol. Subjects trained regularly at competitive level for at least eight years (seven to eight swim sessions and 3–4 gym sessions per week the months before data collection with a mean swimming volume of 40-km per week) and took no drugs or medicine during the study. Mean performance in 400-m front crawl swimming was determined the week before testing (4:22 ± 0:11-s), corresponding to 81% of the short course world record. All swimmers were familiar with the swimming pool exercise testing procedures. The swimmers were instructed to refrain from intense training sessions at least 24 h before the experimental sessions and to retain their normal nutritional habits. All subjects or their parents/guardians (when appropriate) signed an informed consent form prior to participation in the research. The study was approved by the local University Ethical Committee in Human Research from São Paulo State University (UNESP—CAAE:02402512.7.0000.5398) and conducted in accordance with the 1975 Declaration of Helsinki.

### 2.3. Procedures

Tests were conducted at similar time of the day (±2 h) for each swimmer in order to minimize the circadian effect on performance [28] and in separate days (with at least 24 h of rest between tests) in a 25-m swimming pool with the water temperature at 28.2 °C. Body composition was assessed with Tanita BC-543 (Tokyo, Japan) and all tests were swum in front crawl. A standardize warm-up of 600-m aerobic swim of low to moderate intensity was completed in every testing session. During the IST and CT, swimming velocity was controlled through a visual pacer (TAR. 1.1, GBK-electronics, Aveiro, Portugal), with flashing lights on the bottom of the pool, helping swimmers to keep up the predetermined swimming velocity. Split times over 50-m were determined and used by two investigators positioned at 7.5 and 17.5-m of the swimming pool to control athletes’ swimming pace. Within a 10-day period, each subject was asked to complete the following tests.

#### 2.3.1. Maximal Lactate Steady State

Subjects performed, in different days, 30 min constant swimming velocity at 90 and 95% of MAV. Each swimmer was asked to maintain the pre-established swim pace for as long as possible. The test was interrupted when the swimmer could no longer match the required swimming velocity. Each subject was stopped 30-sec every 400-m for blood sample collection determined in fingertip using the Lactate Pro portable analyzer (Arkray, Kyoto, Japan). MLSS was defined as the highest BLC that increased by no more than 1 mmol.L^−1^ during the final 20-min of a 30-min CT [29]. When this criterion was not accomplished, the test was stopped. MLSS_v_ was the swimming velocity associated with MLSS.

Rate of perceived exertion (RPE) was determined in a 6 to 20 scale [30] by verbal indication of the swimmers in the 30-sec stop every 400-m, while blood sample was being collected.

According to the proposal of Craig and Pendergast [31], stroke rate (SR) was calculated for each cycle using the equation (SR = 60/stroke duration) and expressed in cycles per minute (cycles min^−1^). Stroke length (SL) was determined with the equation (SL = V/SR/60) and expressed in meters per cycle (m-cycle^−1^).

SR was measured from three stroke cycles taken in the middle of the pool for every 50 m, SR was measured from three stroke cycles taken in the middle of the pool for every 50 m and averaged based on 100-m distance during the last 20-min swim for the CT tests and the last 100 m of each step for the IST.

The average of heart rate (HR) values collected during the last 20 min of MLSS were determined with Polar Sport Tester (S410), with frequency every 5 s during tests.

#### 2.3.2. Incremental Step Test

Swimmers T_200_ (performance time in 200-m front crawl) was assessed in formal competition with a maximum distance of 2 months for the determination of the IST swimming velocities. Afterwards, in a separate session, swimmers completed 7 × 200-m front crawl IST [10]. All steps started each 5-min, the first one at 70% of T_200_ and the subsequent with a 5% increment. At rest, immediately after each step and at the end of the tests, RPE and BLC were recorded. HR, SR, and SL were measured throughout the test.

The BLC values were registered, and the results were plotted against the respective swimming velocities using Lactate-E software [32]. LT was determined according to the log-log methodology (LT_log-log_), the velocity at which lactate increased exponentially when the log BLC is plotted against the log swimming velocity [12]. LT was also measured according to Dmax methodology (LT_Dmax_) as the maximal perpendicular distance of the lactate curve from the line connecting the start with the endpoint of the lactate curve [13].

vLT_Dmax_ and vLT_log-log_ were the swimming velocities associated to both LT methodologies. V_4_ was considered as the swimming velocity eliciting a lactate concentration of 4 mmol.L^−1^ through linear interpolation [14]. Vmax was assumed as the swimming velocity performed in the last repetition of the IST (100% T_200_).

### 2.4. Statistical Analysis

The data are expressed as the mean ± standard deviation (SD). The normality of the distributions was assessed with the Shapiro–Wilk test, parametric statistical procedures were selected. Linear regression models between swimming velocities in continuous test (MLSSv) and IST (V_4_, vLT_Dmax_ and vLT_log-log_) were computed. Trendline equation, determination coefficient (R^2^), and standard error of estimation (SEE) were calculated. Comparisons of swimming velocities were evaluated using standardized differences with combined variance, derived from the *M* and *SD* of each variable, with 95% confidence intervals. Paired-samples t-test was used to compare swimming performance markers using standardized differences with combined variance, derived from the *M* and *SD* of each variable, with 95% confidence intervals. The statistical limits for the effect sizes Cohen’s *d* [33] were trivial (0–0.2), small (0.2–0.6), moderate (0.6–1.2), large (1.2–2), very large (2–4), and extremely large (>4) [34]. The variance analysis (ANOVA) was used to verify the differences between swimming velocities, the magnitude of the differences was evaluated by eta square (small 0.01 ≤ $\eta_{p}^{2}$ < 0.06), moderate (0.06 ≤ $\eta_{p}^{2}$ < 0.15), or large ($\eta_{p}^{2}$ ≥ 0.15) [33]. The post-hoc Bonferroni test was also performed in order to verify which pairs of means were significantly different (*p* < 0.05). Bland–Altman plot [35] was used to assess the agreement between MLSSv, vLT_log-log_, vLT_Dmax_, and V_4_ showing the bias and the limits of agreement. Also, the concordance correlation coefficient (CCC) was performed using the Lin [36] approach with MedCalc^®^ v11.1.1.0 (2009) software. The CCC (*ρ*c) contains a measurement of precision ρ and accuracy (*ρ*c = *ρ* Cb): where ρ is the Pearson correlation coefficient, which measures how far each observation deviates from the line of best-fit and is a measure of precision, and Cb is a bias correction factor that measures how far the best-fit line deviates from the 45° line through the origin and is a measure of accuracy. Data analysis was performed using the Statistical Package for Social Sciences (SPSS 25.0, SPSS. Inc., Chicago, IL, USA).

## 3. Results

All the fourteen swimmers were able to perform the 30-min constant swimming at 90% of MAV within the criteria established to assume the MLSS and stopped their CTs at an intensity equal to 95% of MAV because of exhaustion. MLSSv (1.36 ± 0.06 m∙s^−1^), was significantly lower compared to Vmax (1.53 ± 0.07 m∙s^−1^; 112% MLSSv) and MAV (1.51 ± 0.07 m∙s^−1^; 111% MLSSv). Vmax and MAV were not significantly different (*p* > 0.05). vLT_Dmax_ (1.40 ± 0.06 m∙s^−1^; 103% MLSSv), V_4_ (1.36 ± 0.07 m∙s^−1^; 100% MLSSv), and vLT_log-log_ (1.34 ± 0.06 m∙s^−1^; 99% MLSSv) were not significantly different to MLSSv. In Table 1 is presented the comparative analysis between the different swimming velocities.

**Figure 2:**
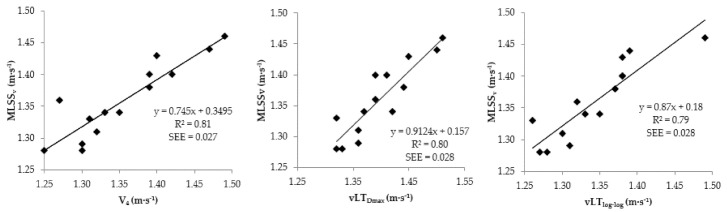
Linear regression of MLSS_v_ on V_4_, vLT_log-log_, and vLT_Dmax_ with standard error of estimate (SEE).

Regression analysis between MLSSv and V_4_ revealed adjusted r^2^ value of 0.81 with a SEE of 0.027, in spite of standardized residuals remaining within the 95% confidence interval limits, indicating a fairly good estimation model. The linear regressions with r^2^ and SEE values between MLSSv, V_4_, vLT_log-log_, and vLT_Dmax_ are presented in Figure 2.

**Figure 3:**
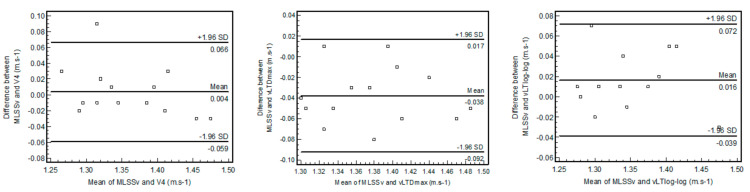
Bland-Altman plot showing the bias and limits of agreement between MLSSv, vLT_Dmax_, and vLT_log-log_.

The agreement between MLSSv and V_4_ is shown in Figure 3. The 95% limits of agreement (Loa) ranged from −0.059 to 0.066. Although the bias was 0.004 and there was no relation between the difference and the mean of the parameters there were somewhat wide limits of agreement (±5.1%). The bias between MLSSv and vLT_log-log_ was −0.016 and the Loa ranged between −0.039 and 0.072, representing a variation of ±4.1% of MLSSv. There was not a significant trend between the difference and the mean of the two measures. The MLSSv was overestimated by the vLT_Dmax_ with a bias of −0.038 with somewhat wide Loa ranging between −0.092 and 0.017, representing a variation of ±4.1% of MLSSv. There was not a significant trend between the difference and the mean of the two measures.

CCC of the methods are shown in Table 2, where <0.90 indicates a poor strength of agreement between the methods.

In IST, it was observed that swimmers tend to neglect SL with the purpose of maintaining the pre-establish swimming velocity throughout all the 7 × 200-m repetitions. Above 85% T_200_, a stroking efficiency breakpoint was observed in all well-trained swimmers, SR and SL in the fourth repetition in the IST (85% of T_200_) (respectively 33.88 ± 3.89 cycles min^−1^ and 2.50 ± 0.32 m cycle^−1^) were significantly different (*p* < 0.01) compared to the fifth repetition, at 90% T_200_ (respectively 36.77 ± 3.20 cycles min^−1^ and 2.40 ± 0.23 m cycle^−1^). SL in the third IST repetition (80% of T_200_ = 2.59 ± 0.31 m cycle^−1^) was significantly higher compared to the fourth and also SL at MLSSv (2.54 ± 0.33 m cycle^−1^; *p* < 0.01). 85% T_200_ (1.36 ± 0.05 m∙s^−1^) was not significantly different from MLSSv (*p* > 0.05), ES was trivial (0.07), and a close relationship between stroke and physiological markers was observed between both, presented in Table 3.

Although not significantly different, in HR the *p* value and Cohen’s *d* revealed values close to significant differences. MLSS ranged between 2.6 and 7.1 mmol L^−1^ and mean LT_D-max_ (5.1 ± 0.7 mmol L^−1^; range 3.9 and 6.2) was not significantly different from MLSS, however, LT_log-log_ (3.8 ± 0.7 mmol.L^−1;^ range 2.6 and 4.5) was lower (*p* < 0.01). Mean RPE during MLSS test was 13.5 ± 1.5, associated to “somewhat hard” with values ranging from 11 to 16 and in the fourth repetition in IT (85% of T_200_) from 12 to 15.

## 4. Discussion

The purpose of this study was to determine if the velocities associated with different lactate parameters determined from IST are equivalent to MLSSv determined from a CT. Additionally, we intent to ascertain if the stroke and physiological parameters, representative of MLSS, are similar to those obtained during the IST. The first main finding in the present study was that MLSS can be determined in well-trained swimmers with only two to three attempts of 30-min constant swimming velocity performed in different days assuming the first swimming test at 90% MAV, the swimming velocity at which athletes participating in our study achieved the MLSS. Second, although it is interesting to speculate if an IST provides reliable indicators of MLSS, we verified that from a practical perspective, daily training, some outputs may be useful for swimmers and coaches, but an accurate evaluation of MLSS is only possible through the traditional methodology, a CT.

Although there was not a significant statistical difference between MLSSv, V_4_, vLT_log-log_, and vLT_Dmax_, there was a poor CCC (<0.90) between the MLSSv and the other three lactate indexes, which precludes the use of these measures interchangeably. V_4_ did not present a bias when compared with MLSSv determined in a CT, however, Bland–Altman analysis showed somewhat wide limits of agreement (±5.1%), which in a practical point of view can represent meaningful differences. For example, a swimmer with a MLSSv of 1.36 m∙s^−1^ can have a V_4_ of 1.41 or 1.32 m∙s^−1^, which represents a difference of 4 sec each 100 m. Regarding vLT_log-log_, it underestimated MLSSv by 0.02 m∙s^−1^ with limits of agreement of ±4.1%. Conversely vLT_Dmax_ was consistently higher than MLSSv by 0.04 m∙s^−1^, with Bland–Altman analysis also showing limits of agreement of ±4.1%.

Previously, a MLSSv of 1.22 ± 0.09 m∙s^−1^, representing 88.9 ± 3.3% of MAV, was found in eleven male well-trained competitive swimmers [37]. Also, Baron et al. [29] in ten well-trained competitive swimmers showed that MLSS corresponded to the velocity a swimmer spontaneously chooses during the first 15 min of a 2-h test. These authors also observed a MLSS_v_ of 1.22 ± 0.14 m∙s^−1^, corresponding to 86.5 ± 5.1% of MAV (1.41 ± 0.12 m∙s^−1^). Later, Espada, and Alves [38] also observed a MLSSv of 1.34 ± 0.06 m∙s^−1^ corresponding to 89.7 ± 1.7% of MAV. However, another study conducted with twelve middle-distance and long-distance male swimmers showed that the stroke parameters and BLC were significantly different between MLSS_v_ (1.22 ± 0.05 m∙s^−1^; 88.6 ± 1.1% of MAV) and 102.5% MLSS_v_ (1.25 ± 0.04 m∙s^−1^; 91.3 ± 1.1% MAV) [6].

In the present study, swimmers were able to perform 30-min at 90% of MAV, which can be related with the level of the swimmers participating in our study since to our best knowledge this is the first study to compare the speed, physiological and stroke parameters determined from the IST and CT methods in well-trained swimmers with MLSSv above 1.35 m∙s^−1^. However, in previous research some swimmers were able to complete 30-min at 90% MAV, although, without meeting MLSS criteria. For example Dekerle et al. [37] reported that five swimmers could complete the 30-min swim but increased their BLC values by more than 1 mmol L^−1^ between the 10th min (4.4 ± 1.6 mmol L^−1^) and the 30th min (5.9 ± 1.9 mmol L^−1^) and in Pelarigo et al. [6] study, the 102.5% MLSS intensity was maintained without exhaustion in the 30-min test but the criteria to be considered MLSS was also not accomplished.

Lower MLSS values were found in studies conducted with swimmers, which can be attributed to the lower MLSSv values compared to our study. Also lower values were evident in rowers (3.1 ± 0.5 mmol L^−1^) but higher in cyclists (5.4 ± 1.0 mmol L^−1^) and speed skaters (6.6 ± 0.9 mmol L^−1^) [17]. In cycling, Van Schuylenbergh et al. [39] found a significant correlation between MLSS workload and V_4_, which led to the indication that LT (determined from Dmax methodology) was closely correlated with MLSS power (r = 0.72). Though, these authors point out that the validity of MLSS predicted from an IST must be verified by a 30-min constant load test. In swimming, a significant correlation between LT and V_4_ (r = 0.90; *p* < 0.01) [40] was found and a study conducted with five male long-distance swimmers and eight triathletes showed that vLT (determined from Dmax methodology), although higher, was not significantly different than MLSS_v_ (1.18 ± 0.08 m∙s^−1^ and 1.13 ± 0.08 m∙s^−1^, respectively) [41]. Conversely, Fernandes et al. [9] determined V_4_ and V_8_ (swimming velocity at 8 mmol.L^−1^) by linear interpolation or extrapolation of the lactate BLC vs. velocity curve in a IST (respectively 1.20 ± 0.15 and 1.30 ± 0.17 m∙s^−1^), with both being significantly higher than MLSSv (1.09 ± 0.14 m∙s^−1^) in seventeen long-distance swimmers. Although previous research reported that the 7 × 200 protocol is sensible to different training regimens [42] our results seem to confirm that the parameters derived from this protocol cannot be used interchangeably with the MLSS gold standard determination protocol.

Furthermore, our results also confirm that it is impossible to link the true MLSS to a fixed lactate concentration as it was previously pointed [5], because MLSS ranged from 2.6 to 7.1 mmol.L^−1^. It should be noted that MLSS in swimming is affected by brief interruptions in exercise that are necessary for blood sampling. On the other hand, it was recently indicated that 30 to 45-sec passive recovery between 10 × 200-m swimming repetitions enables steady BLC, oxygen uptake and HR similar to MLSS [23]. However, we must acknowledge that the dynamic interaction between the rates of muscle lactate production, lactate efflux from muscle to blood, and lactate clearance/metabolism both within muscle and from the blood by other organs [43], means that a steady-state in BLC need not imply the existence of a bioenergetic steady-state in contracting skeletal muscle [19]. These authors indicated that BLC per se, is neither an appropriate nor a sufficiently sensitive metric to enable a confident assessment of whether a specific velocity or power output may be sustainable in a metabolic steady-state muscle.

Pelarigo et al. [6], found a MLSS_v_ of 1.22 ± 0.05 m∙s^−1^ and a T_200_ of 1.45 ± 0.05 m∙s^−1^ which means that MLSSv corresponded to around 84% of T_200_. Additionally, another study indicated that the decrease in SL started above 85% of MAV [37] and Fernandes et al. [9] observed that SR was different across the 7 × 200-m particularly after the 4th repetition. It was previously stressed that MLSS could represent an intensity to develop aerobic endurance and perform technical work of very-high-standard quality [29,37], fact that was confirmed by Pelarigo et al. [6] research where the MLSS (3.28 ± 0.97 mmol.L^−1^) was significantly lower than BLC at 102.5% MLSSv (4.59 ± 1.36 mmol.L^−1^) and the SR was maintained in MLSSv between 10th and 30th minute and significantly increased at 102.5% MLSS, contrary to SL, maintained during the 30 min swam at MLSS but significantly decreased at 102.5% MLSS_v_.

Our study revealed that during IST, the SR and SL at 85% of T_200_, the fourth repetition, were closely related to those observed at MLSS throughout the 30 min CT and tend to represent a boundary of the swimming efficiency, showing that the transition from the heavy to the severe intensity domain is not only related to swimming velocity, RPE and BLC, but also to stroke parameters. The values we measured in MLSS (SR 32.8 ± 4.1 cycles.min^−1^/SL 2.54 ± 0.33 m.cycle^−1^) in well-trained swimmers were also higher compared to previous studies, such as Dekerle et al. [37] (27.7 ± 2.2 cycles.min^−1^; 2.64 ± 0.32 m.cycle^−1^) or Pelarigo et al. [6] (30.9 ± 3.4 cycles.min^−1^; 2.47 ± 0.2 m.cycle^−1^), fact that we consider associated to the level of swimmers participating in the different studies.

Baron et al. [44] verified that during exercise performed at MLSS, exhaustion occurred while physiological reserve capacity still existed, but in association with an increase in the RPE, as predicted by the central governor model. This research team added that exercise termination may be induced by an integrative homoeostatic control of the peripheral physiological system specifically to ensure the maintenance of homeostasis. Demello et al. [45] indicated that LT occurs at a feeling of “somewhat hard” and “hard” from the RPE perspective, with values ranging from 12.9 to 13.6. Our results are in line with these previous observations, although the CT values ranged from 11 to 16 and in the 4th repetition, in IST (85% of T_200_) from 12 to 15, fact that led us to agree with previous literature stating that the use of an absolute RPE value to prescribe exercise intensity is unwise [46] because of the fairly large between-subject variability. Also, Potteiger and Weber [47] investigated RPE during incremental and constant intensity exercise and concluded that RPE cannot be used as a particularly accurate marker of exercise intensity.

This study presents some limitations since we did not consider the maturation of the swimmers or their distance specialty, which may be relevant to the training methodological framework. We also did not evaluate the potential differences regarding sexes since we only tested male athletes, neither compared results in short- and long-course swimming pools, factors that make it impossible to generalize our results to the whole swimming community. Tracking individual responses during the swimming process is crucial for training prescription and adjustments as inter-individual differences are significant in well-trained athletes. Future research should consider different swimmers’ level, gender, and the comparison between CT and IST in long-course swimming pool (50-m), with swimming velocities increment of 2.5%, as implemented by Pessôa-Filho et al. [48]. The possible measurement of gas exchange could be useful to better understand the physiological, metabolic, and stroke parameters pathways associated to CT and IST, as well as more accurately measure LT and MLSS, understanding the athletes holistic fatigue associated to the gold standard, not only from the BLC perspective.

## 5. Conclusions

From a practical application perspective, the main findings of this study show that in well-trained swimmers MLSSv can be estimated with a maximum two to three 30-min constant swimming bouts performed in different days starting at 90% of MAV and elevating or decreasing that exercise intensity in subsequent bouts. This procedure is more practical and less time consuming than protocols previously suggested which could be important for coaches and athletes in daily swimming practice.

Moreover, the 90% of MAV or 85% of T_200_ may be considered aerobic power zones, where high-quality technique training may occur. In fact, stroke parameters achieved during MLSSv are very closely related to the fourth repetition of the IST and represent not only a physiological, but a mechanical boundary above which athletes achieve fatigue and the swimming technique starts to deteriorate.

Performing an IST in well-trained swimmers can be very useful for practice because it provides several useful indicators, nevertheless, this methodology should be used with caution. Also, the level of the swimmers seems to decisively influence the obtained results, this fact should be carefully examined in future research.

V_4_, vLT_Dmax_, and vLT_log-log_ were not statistically different from MLSS_v_, nevertheless, it is our understanding that both the IST and CT in well-trained swimmers provide useful indexes of aerobic potential, but they cannot be used interchangeably for MLSS_v_ determination. The direct determination of MLSS_v_, with CT testing procedure, remains the more accurate for evaluation and prescription of swimming training and for research purposes.

## Figures and Tables

**Table 1:** Comparative analysis between different swimming velocities.

		Anova *One-way*			
Tests	M ± SD	F	*p*	$\eta_{p}^{2}$	Post-Hoc Test Bonferroni
Vmax (m∙s^−1^)	1.53 ± 0.07	22.20	0.00	0.58	MAV (1.000); vLT_Dmax_ (0.000); MLSS_v_ (0.000); V_4_ (0.000); vLT_log-log_ (0.000)
MAV (m∙s^−1^)	1.51 ± 0.07				Vmax (1.000); vLT_Dmax_ (0.000); MLSS_v_ (0.000); V_4_ (0.000); vLT_log-log_ (0.000)
vLT_Dmax_ (m∙s^−1^)	1.40 ± 0.06				Vmax (0.000); MAV (0.000); MLSSv (1.000); V_4_ (1.000); vLTlog-log (0.452)
MLSS_v_ (m∙s^−1^)	1.36 ± 0.06				Vmax (0.000); MAV (0.000); vLT_Dmax_ (1.000); V_4_ (1.000); vLT_log-log_ (1.000)
V_4_ (m∙s^−1^)	1.36 ± 0.07				Vmax (0.000); MAV (0.000); vLT_Dmax_ (1.000) MLSS_v_ (1.000); vLT_log-log_ (1.000)
vLT_log-log_ (m∙s^−1^)	1.34 ± 0.06				Vmax (0.000); MAV (0.000); vLT_Dmax_ (0.452) MLSS_v_ (1.000); V_4_ (1.000)

M ± SD, mean ± standard deviation; Vmax, swimming velocity performed in the last repetition of the incremental step test; MAV, maximal aerobic velocity; vLT_Dmax_, swimming velocity associated to lactate threshold determined from Dmax methodology; MLSSv, swimming velocity associated to maximal lactate steady state; V_4_, swimming velocity eliciting a lactate concentration of 4 mmol.L^−1^; vLT_log-log_, swimming velocity associated to lactate threshold determined from log-log methodology. Note: Anova one-way F, *p* and $\eta_{p}^{2}$ related to all swimming velocities. Significant differences between swimming velocities (*p* < 0.05) are observed with post-hoc test.

**Table 2:** Concordance correlation coefficient between MLSSv and the three lactate indexes determined in the incremental step test.

	CCC	Precision	Accuracy
V_4_ (m∙s^−1^)	0.88	0.90	0.98
vLT_log-log_ (m∙s^−1^)	0.86	0.89	0.96
vLT_Dmax_ (m∙s^−1^)	0.74	0.90	0.83

CCC, concordance correlation coefficient; V_4_, swimming velocity eliciting a lactate concentration of 4 mmol.L^−1^; vLT_log-log_, swimming velocity associated to lactate threshold determined from log-log methodology; vLT_Dmax_, swimming velocity associated to lactate threshold determined from Dmax methodology.

**Table 3:** Mean and standard deviation of performance markers during MLSS test and fourth repetition in the incremental step test, at 85% T_200_.

*Variable*	MLSSv	85% T_200_	*t*	*p*	Cohen’s *d*
HR (beats.min^−1^)	174.2 ± 7.0	169.5 ± 6.2	1.841	0.077	−0.70
SR (cycles.min^−1^)	32.76 ± 4.07	33.88 ± 3.89	−0.749	0.461	0.28
SL (m.cycle^−1^)	2.54 ± 0.33	2.50 ± 0.32	0.269	0.790	−0.10
BLC (mmol.L^−1^)	4.84 ± 1.53	4.83 ± 0.94	0.015	0.988	−0.01
RPE (6–20 scale)	13.50 ± 1.50	13.28 ± 0.72	0.479	0.637	−0.18

MLSSv, swimming velocity associated to maximal lactate steady state; T_200_, performance time in 200-m front crawl; HR, heart rate; SR, stroke rate; SL, stroke length; BLC, blood lactate concentration; RPE, rate of perceived exertion. ES are considered trivial (0–0.2), small (0.2–0.6), moderate (0.6–1.2), large (1.2–2), very large (2–4) and extremely large (>4) (Cohen’s *d*). In all cases *p* > 0.05 represent no significant statistical differences.

## Data Availability

The data that support the findings of this study are available from the corresponding and first authors (joanareis@fmh.ulisboa.pt and mario.espada@ese.ips.pt), upon reasonable request.
